# Peripheral Perfusion Index: An Adjunct for the ED Triage or a Powerful Objective Tool to Predict Patient Outcomes?

**DOI:** 10.3390/jcm14134616

**Published:** 2025-06-29

**Authors:** Veysi Siber, Serdal Ateş, Tuba Şafak, Ebru Güney, Aycan Uluçay, Şeyda Gedikaslan, Sinan Özdemir, Muhammed Sezai Bazna, Michal Pruc, Pawel Patrzylas, Lukasz Szarpak, Burak Katipoglu, Ahmet Burak Erdem

**Affiliations:** 1 Department of Emergency Medicine, Ministry of Health Ankara Etlik City Hospital, Ankara 06170, Türkiye; veysiber.ss@gmail.com (V.S.); allonsyebru@gmail.com (E.G.); aycanulucay15@gmail.com (A.U.); burak44katipoglu@gmail.com (B.K.); drabe0182@gmail.com (A.B.E.); 2 Ankara Training and Research Hospital, University of Health Sciences, Ankara 06230, Türkiye; 3 Giresun State Hospital, Giresun 28100, Türkiye; gedikaslanseyda@gmail.com; 4 Düzce Atatürk State Hospital, Düzce 81010, Türkiye; ozdemirs32@gmail.com; 5 Departmant of İntensive Care Unit, Ministry of Health Ankara Etlik City Hospital, Ankara 06170, Türkiye; 6 Department of Clinical Research and Development, LUXMED Group, 02-678 Warsaw, Poland; 7 Institute of Biological Science, Collegium Medicum, The John Paul II Catholic University of Lublin, 20-950 Lublin, Poland; 8 Henry JN Taub Department of Emergency Medicine, Baylor College of Medicine, Houston, TX 77030, USA; 9 Institute of Medical Science, Collegium Medicum, The John Paul II Catholic University of Lublin, 20-950 Lublin, Poland

**Keywords:** peripheral perfusion index, emergency department triage, Emergency Severity Index, risk stratification, predictive biomarker, patient disposition

## Abstract

**Background/Objectives**: Accurate and timely triage is essential for optimizing clinical outcomes and resource allocation in emergency departments (EDs). The Peripheral Perfusion Index (PPI), a non-invasive and objective parameter derived from pulse oximetry, may offer added value in early risk stratification. This study aimed to analyze the correlation between the PPI measured at triage and at Emergency Severity Index (ESI) levels, as well as to determine if the PPI may function as a predictive tool to facilitate early risk identification before patient disposition. **Methods**: In this prospective cross-sectional study, adult ambulatory patients presenting to a tertiary care ED were enrolled. At triage, PPI and standard vital signs were recorded, and patients were classified using the five-level ESI system. The diagnostic performance of PPI and ESI in predicting ED discharge was assessed using receiver operating characteristic (ROC) curve analysis, with comparative evaluation performed via DeLong’s test. **Results**: Lower PPI values were consistently associated with higher ESI acuity levels and more intensive care requirements. Patients who were discharged had significantly higher median PPI values (4.0) compared to those admitted to wards (2.1) or intensive care units (1.9). PPI also distinguished survivors from non-survivors (median PPI: 3.60 vs. 1.15). ROC analysis showed that the PPI demonstrated a good discriminative capacity for forecasting ED discharge, equal to the efficacy of ESI (AUC: 0.926 vs. 0.903; *p* < 0.001). **Conclusions**: The PPI could improve post-triage risk classification and enhance current triage techniques like ESI, especially in cases of unclear or borderline presentations, but further validation in prospective trials is required.

## 1. Introduction

Triage in emergency departments (EDs) is a critical process that enables the classification of patients based on the urgency of their clinical condition, with the aim of utilizing available resources in the most efficient manner. Furthermore, accurate triage is directly associated with improved patient outcomes, including reduced in-hospital mortality and length of stay, particularly in resource-constrained environments where rapid clinical decision-making is vital. Triage plays a key role in reducing this burden by enabling the early identification and prompt management of clinically unstable patients [1]. In Türkiye, the total number of ED visits was approximately 91 million in 2013, and this figure nearly doubled over the following decade, reaching approximately 151 million by 2023 [2]. In Tuürkiye, the triage system currently in use is based on a color-coded scale. On a global scale, people commonly implement several structured triage systems, with the Emergency Severity Index (ESI) being the most widely adopted. The ESI is a five-level ordinal scale in which the urgency of the patient’s clinical condition decreases progressively from level 1 to level 5. ESI level 1 includes patients requiring immediate life-saving interventions. In general terms, patients classified as ESI levels 1 or 2 are considered non-deferrable and require immediate evaluation, while those in levels 4 and 5 are deemed to be lower acuity and may wait safely or be assessed in a fast-track unit [3,4,5].

Despite the ESI being one of the most prevalent triage systems globally, it is significantly dependent on the clinical experience and observational judgment of the triage provider, resulting in variations in classification. Conversely, established five-level triage systems such as the Modified Rapid Emergency Medicine Score (mREMS), the Canadian Triage and Acuity Scale (CTAS), the Manchester Triage System (MTS), and the Australasian Triage Scale (ATS) [6,7,8] are systematic, complaint-oriented algorithms. They integrate objective criteria, including vital sign thresholds, injury mechanisms, hemorrhage risk, and pain intensity, alongside comprehensive modifiers to facilitate decision-making. For example, the mREMS provides a score ranging from 0 to 26, and patients with a score above 13 are considered to be at high risk for mortality [9,10]. The Peripheral Perfusion Index (PPI) is a non-invasive parameter measured via pulse oximetry that reflects peripheral tissue perfusion by assessing pulsatile blood flow. It represents the ratio between pulsatile (arterial) and non-pulsatile (static) blood flow components, expressed as a percentage. PPI values typically range from 0.02% to 20%. It can be utilized in a variety of clinical contexts, from the early detection of shock to the assessment of fluid responsiveness [11]. Moreover, the PPI allows for continuous monitoring, which enhances its clinical applicability. The PPI is not intended as the primary triage measure but rather as a supplementary complement to current triage instruments. However, its non-invasive nature, rapid measurement, and ease of use increase its potential for integration as a supportive tool within triage systems in the emergency department setting. Indeed, data on its implementation in routine ED triage workflows remain scarce, and evidence regarding its correlation with validated triage scales, such as the ESI, is limited.

This study aimed to evaluate the correlation between the PPI measured at triage and the triage severity level determined by the ESI, as well as determine whether the PPI offers prognostic information regarding patient outcomes, including the necessity for hospitalization or the risk of short-term mortality.

## 2. Materials and Methods

### 2.1. Study Design

This manuscript was prepared in accordance with the STROBE (Strengthening the Reporting of Observational Studies in Epidemiology) guidelines for cross-sectional studies to ensure transparent and comprehensive reporting.

This prospective cross-sectional study was conducted following approval from the Ethics Committee of the Ankara Etlik City Hospital (Approval No. AEŞH-BADEK-2024-602). The study was carried out in the ED of Ankara Etlik City Hospital, which receives approximately 50,000 patient visits per month. Inclusion criteria were: (1) age ≥ 18 years; (2) presentation to the ED for non-traumatic complaints. The exclusion criteria were as follows: (i) Patients who did not provide informed consent; (ii) Patients who arrived at the ED via ambulance; (iii) Patients with a history of alcohol or other sedative/narcotic substance use; (iv) Patients who later withdrew informed consent; (v) Patients in whom a PPI measurement could not be performed (e.g., peripheral vascular disease, autonomic neuropathy, or central hypothermia with core body temperature < 36 °C); (vi) Patients with trauma or known malignancy. Moreover, only ESI-1 patients who had achieved transient stability after initial resuscitative interventions and allowed for safe measurement of the PPI within 15 min were included. Patients requiring immediate, uninterrupted interventions were excluded. Data collection occurred during a continuous four-week duration, with sampling performed during 60 of the 84 total shifts (morning, evening, and night) across both weekdays and weekends. Eligible patients were enrolled consecutively during staffed shifts by trained research personnel. Despite staffing limitations, the schedule was designed to accommodate fluctuations in patient flow over various hours and days. On average, 30 to 50 eligible patients were enrolled per shift, depending on load and staffing availability.

### 2.2. Variable Measurements and Emergency Severity Index Score

Hospitals in Türkiye perform triage coding using a color scale system. In addition, triage personnel routinely recorded the patient’s age, sex, triage code, and basic vital parameters. The vital signs assessed included heart rate (HR), respiratory rate (RR), systolic blood pressure (SBP), diastolic blood pressure (DBP), oxygen saturation (SpO_2_), and body temperature.

All patients were assessed by physicians of equal training and experience who had completed a 10-h theoretical and practical triage education program. Patient selection and ESI scoring were performed in accordance with recommendations from the existing literature. ESI classification was conducted based on the manual triage system as follows:

Level 1: Patient requiring immediate life-saving intervention

Level 2: High-risk or emergent patient

Level 3: Patient classified as urgent but able to wait safely

Level 4: Non-urgent patient

Level 5: Patient not requiring any resources in the ED [12].

Blood pressure was measured using a non-invasive technique after the patient had rested for 5 min in the supine position. Respiratory rate was determined by visually counting chest movements for one full minute while the patient remained silent and motionless in a lying position.

HR, PPI, and SpO_2_ measurements were obtained after patients remained in the supine position for 5 min. As specified in the device’s user manual, measurements were performed using the MASIMO MODEL: RAD-97 patient monitor (Masimo Corporation, Irvine, CA, USA), with a pulse oximeter probe attached to the second or third finger of the patient’s left hand. We recorded the measurement values either after achieving a stable reading on the monitor or after a 15-s interval. This is the same device and procedure employed to acquire standard SpO_2_ and HR measurements during triage. Consequently, the PPI value was documented concurrently with SpO_2_ and HR without necessitating supplementary equipment or interrupting the regular triage process. Blood pressure, respiration rate, and several parameters were simultaneously measured using normal non-invasive methods. The MASIMO RAD-97 monitor calculates PPI as the ratio of pulsatile arterial flow in peripheral tissues (AC: arterial circulation) to non-pulsatile or static blood flow (DC: direct circulation), expressed as AC/DC × 100. The device provides values ranging from 0.02 to 20. The Masimo RAD-97 monitor used in this study has been previously validated for clinical accuracy in both adult and pediatric populations [11]. Calibration was confirmed according to manufacturer standards prior to the study period. All measurements were conducted by trained triage personnel who received dedicated instruction on probe placement and data recording to minimize inter-observer variability.

To improve reliability, the PPI was measured after a 5-min stabilization period, and only recordings with stable waveform and signal quality index ≥ 90% were accepted. If signal stability was not achieved within 15 s, the measurement was repeated. Inter-rater reliability for PPI measurements was tested on a subset of 50 patients using the intraclass correlation coefficient (ICC), which showed excellent agreement (ICC > 0.90). Patient outcomes were analyzed and categorized as discharge, ward admission, or admission to the ICU. Mortality was assessed within 30 days following the initial ED presentation.

### 2.3. Outcome Measures

The primary outcome of the study was to assess the strength of a correlation between the PPI and the ESI, with the aim of evaluating the potential utility of the PPI as an objective and real-time triage parameter in emergency settings. Triage nurses were blinded to PPI values, and the PPI was not used to determine ESI assignments.

The secondary outcomes were: (i) the predictive value of the PPI in determining emergency department disposition (discharge vs. hospital or ICU admission) and (ii) the association between PPI results and 30-day mortality. Hospital admission was defined as any transfer to a monitored unit (ward or ICU) directly from the ED, while mortality referred to all-cause death within 30 days of ED presentation, verified by electronic health records and national mortality registries. All data were prospectively entered into a secure digital database by the research team. Data accuracy was verified via dual entry for 10% of cases and random auditing. Investigators analyzing clinical outcomes and performing ROC analysis were blinded to the PPI values at the time of triage.

### 2.4. Statistical Analysis

The sample size was determined to identify a minimal clinically significant difference of 0.03 in AUC between the PPI and the ESI for predicting hospital admission, with α = 0.05 and power = 0.9, necessitating a minimum of 880 participants. Although the primary aim of the study was to assess the correlation between PPI and ESI levels, the sample size was calculated based on the secondary outcome (predictive performance of the PPI for ED disposition) to ensure sufficient power for both objectives. This approach allowed robust evaluation of correlation coefficients and discriminative analyses while maintaining clinical and statistical rigor. To increase statistical power and ensure subgroup analysis validity, we included a final sample of 2539 patients.

Statistical analyses were conducted using SPSS version 27 and Jamovi version 2.5.7. Categorical variables were summarized with frequencies and percentages and compared using the chi-square test. Numerical variables’ normality was evaluated with the Kolmogorov–Smirnov test and histogram plots and reported as medians with interquartile ranges (IQR, 25–75th percentiles). For normally distributed numerical variables, differences between two groups were assessed with a Student’s *t*-test, with variance homogeneity checked via Levene’s test. Non-normally distributed data were compared using the Mann–Whitney U test for two groups and the Kruskal–Wallis test for more than two groups, with post hoc analysis performed using the Dwass-Steel-Critchlow-Fligner (DSCF) test.

To evaluate the diagnostic performance of the PPI and ESI in predicting emergency department discharge, Receiver Operating Characteristic (ROC) curve analysis was employed, calculating threshold values, sensitivity, specificity, positive predictive value (PPV), negative predictive value (NPV), positive likelihood ratio (+LR), and negative likelihood ratio (−LR). The Youden Index determined optimal cut-off points, and the DeLong test compared the diagnostic performance of the two tests. Spearman’s correlation analysis assessed the relationship between the PPI and vital signs. Interobserver agreement for PPI measurements was excellent, with an intraclass correlation coefficient (ICC) of 0.92 (95% CI: 0.88–0.95). Multivariable logistic regression models were constructed to explore the independent predictive value of PPI for admission and mortality, adjusted for age, sex, heart rate, systolic blood pressure, and SpO_2_. Odds ratios (OR) and 95% confidence intervals (CI) were reported. Model discrimination was assessed using the c-statistic, and calibration was evaluated with the Hosmer–Lemeshow test. A *p*-value < 0.05 was considered statistically significant.

## 3. Results

A total of 2539 adult patients were included in the final analysis. The median age of the cohort was 47 years (interquartile range [IQR]: 31–66), and 1346 patients (53.0%) were male. Based on the ESI, patients were stratified into five triage levels: 22 (0.9%) were categorized as ESI level 1, 616 (24.3%) as level 2, 638 (25.1%) as level 3, 620 (24.4%) as level 4, and 643 (25.3%) as level 5.

The median PPI across the study population was 3.60 (IQR: 2.60–4.70). Regarding clinical outcomes, 2084 patients (82.1%) were discharged directly from the emergency department, while 455 (17.9%) required hospital admission—331 (13.0%) to general wards and 124 (4.9%) to intensive care units (ICU). The overall 30-day mortality rate was 0.7% (n = 18). Detailed demographic characteristics, vital signs, and disposition outcomes are summarized in Table 1.

A statistically significant variation in PPI values was observed across ESI categories (*p* < 0.001, Kruskal–Wallis test), as presented in Table 2. Subsequent post hoc analysis using the Dwass–Steel–Critchlow–Fligner (DSCF) test confirmed significant differences between all pairwise ESI level comparisons (all *p*-values < 0.001), indicating a stepwise decline in PPI with increasing clinical acuity.

Similarly, a significant association was identified between PPI values and emergency department (ED) disposition outcomes (discharge, ward admission, ICU admission) (*p* < 0.001, Kruskal–Wallis test). Post hoc comparisons again revealed statistically significant differences across all outcome groups (all *p*-values < 0.001, DSCF test), with lower PPI values corresponding to higher levels of care.

**Figure 1:**
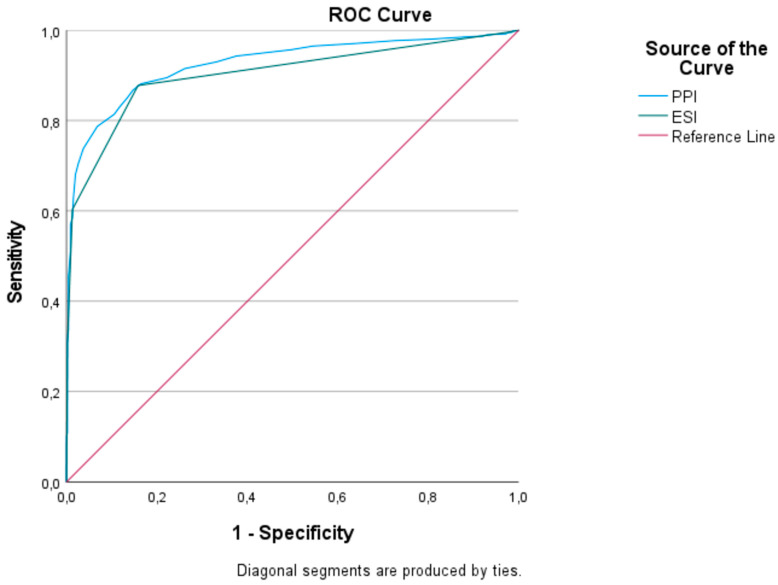
ROC curve of ESI and PPI for predicting emergency department discharge.

The diagnostic accuracy of the PPI and ESI in predicting ED discharge is illustrated in Figure 1. Both indicators demonstrated excellent discriminative performance. The area under the receiver operating characteristic (ROC) curve for the PPI was 0.926 (95% confidence interval [CI]: 0.914–0.937), with a positive likelihood ratio (+LR) of 5.89 and a negative likelihood ratio (−LR) of 0.16. In comparison, the ESI yielded an AUC of 0.903 (95% CI: 0.890–0.915), with a +LR of 5.55 and a −LR of 0.15 (Table 3 and Table 4).

When formally comparing the AUCs using the DeLong test, the PPI was found to be significantly superior to the ESI in predicting discharge outcomes (AUC difference = 0.023, 95% CI: 0.017–0.029; *p* < 0.001). These findings suggest that the PPI offers enhanced diagnostic precision over the ESI and may serve as a valuable objective adjunct in triage-based decision-making. The PPI was found to be correlated with systolic blood pressure, diastolic blood pressure, oxygen saturation, and respiratory rate; however, these correlations were not strong. A Spearman’s rank correlation test revealed a moderate inverse correlation between the PPI and ESI levels (ρ = −0.42, *p* < 0.001), suggesting that lower PPI values were associated with higher acuity.

We conducted a multivariable logistic regression analysis to evaluate the independent predictive value of the PPI for hospital admission, adjusting for age, sex, heart rate, systolic blood pressure, and oxygen saturation. The model demonstrated a significant association between lower PPI values and increased odds of hospital admission (OR: 3.3 × 10^−8^; 95% CI: 6.99 × 10^−11^–1.56 × 10^−5^; *p* < 0.001), thereby establishing the PPI as a reliable independent predictor of clinical deterioration. Additional covariates such as age, sex, HR, SBP, and oxygen saturation (SpO_2_) did not demonstrate statistical significance in the model (Table S1). The results corroborate our non-parametric comparisons and ROC analysis, reinforcing the value of the PPI as an objective marker in triage decision-making.

## 4. Discussion

In our study, we evaluated the initial PPI values and vital parameters of 2539 patients over the age of 18 who presented to the ED on an outpatient basis. Our study demonstrated that lower PPI values were associated with increased clinical deterioration in a severity-dependent manner. Patients who were discharged had significantly higher PPI values compared to those who were not discharged. When hospitalized patients were stratified into ward and ICU admissions, a statistically significant difference in PPI values was also observed between the two groups. Moreover, our study found a statistically significant difference in PPI values between survivors and non-survivors. The median PPI value was 3.60 in survivors, whereas it was significantly lower at 1.15 in patients who died. Again, these findings suggest that the PPI may serve as a clinically predictive parameter. In the study by Kwak et al., it was suggested that the ESI triage system may have limitations in predicting discharge, hospital admission, and mortality due to the lack of support from objective parameters [13]. Van Gendersen et al. reported that the PPI may be useful in predicting complications during perioperative periods [14]. The PPI may serve effectively as a post-triage clinical support instrument, particularly before discharge, by assisting clinicians in identifying patients who seem stable yet may necessitate additional monitoring. In decision-making frameworks, the PPI may function as an element of a System 2 process: an objective, analytical tool that facilitates intentional and critical reasoning following initial triage classification.

It is important to note that triage nurses are largely responsible for prioritizing patients according to acute clinical urgency and aim to measure urgency rather than prognosis. Although our findings indicate that the PPI may be better suitably employed for post-triage risk evaluation, we decided to analyze the PPI as a rapid bedside adjunct for triage. As clinical severity increased from ESI level 5 to level 1, a corresponding decrease in PPI values was observed. Additionally, the PPI cut-off values determined for each of the five ESI triage categories were found to be highly significant. These findings support the potential use of the PPI as an effective triage tool, comparable to the ESI yet based on objective measurement rather than subjective clinical observation and experience, particularly in high-volume EDs with varying staff experience levels. The study conducted by Das et al. [15], which included 367 patients, is among the first in the literature to examine the correlation between the ESI and the PPI. The study found that lower PPI values were associated with an increased likelihood of hospital admission and 30-day mortality. Furthermore, the PPI was also found to be a significant predictor of ESI levels 1 and 2, which represent patients requiring immediate or urgent medical intervention. In our study, a substantially larger patient population was analyzed, and the PPI was found to be a significant predictor across all ESI levels. ROC curve analysis demonstrated that the PPI had a very high diagnostic performance in predicting ED discharge. The elevated AUC for the PPI indicates its precision and capacity to detect nuanced physiological alterations; nonetheless, this does not affect the comprehensive clinical assessment inherent in the ESI paradigm. Using a cut-off value of 2.7 for the PPI, the sensitivity was 87%, specificity was 85%, positive predictive value was 96%, and negative predictive value was 58%. Similar results have been reported in the literature, supporting the consistency of our findings [16,17,18]. However, our application of ROC curves aimed to investigate relative discriminatory performance, rather than to suggest equivalence or superiority in a triage structure.

The observed decrease in PPI values across worsening ESI levels likely reflects progressive peripheral vasoconstriction, which is a compensatory response to central hypovolemia or shock. Our results provide further evidence that perfusion-based markers such as the PPI reflect physiological reserve and may offer advantages over traditional vital signs that often fail to capture early hemodynamic compromise. Although our data seem to indicate that the PPI may provide additional prognostic value alongside conventional triage approaches, PPI in itself may be better suitably employed for post-triage risk evaluation, specifically in identifying patients who seem stable yet may deteriorate following discharge.

### Limitations

Several limitations of this study should be acknowledged. First, this was a single-center investigation conducted in a tertiary care hospital, which may limit the generalizability of our findings to other healthcare settings, particularly smaller or resource-limited emergency departments. Variability in staff experience, triage protocols, and patient demographics across institutions may influence both ESI scoring and PPI measurements. Second, although the study included a large sample size, its cross-sectional design precludes causal inference. While we demonstrated associations between the PPI and clinical outcomes, longitudinal data would be required to establish the predictive value of the PPI over time or to evaluate its response to clinical interventions. Third, we excluded certain patient groups, such as those with trauma, malignancy, substance use, or peripheral vascular disorders, which may introduce selection bias. These exclusions were necessary to reduce confounding but may also limit the applicability of the findings to broader ED populations. Fourth, PPI values may be influenced by external factors, including ambient temperature, probe positioning, peripheral vasoconstriction, and sympathetic tone—all of which were not controlled or standardized beyond routine measurement practices. Although all measurements were performed using the same device and by trained personnel, inter-operator variability in PPI acquisition cannot be completely excluded. Fifth, we assessed mortality within a 30-day follow-up period; however, longer-term outcomes such as 90-day mortality, readmission, or deterioration after discharge were not evaluated, which could provide further insight into the prognostic utility of PPI. Sixth, the distribution of ESI scores in our study does not follow a natural bell curve, as we employed stratified inclusion to ensure analytical balance across categories. This method may limit external generalizability but supports robust intergroup comparisons. Seventh, the PPI is a continuous measure while the ESI is an ordinal five-level system, so the ROC curves could be cautiously interpreted. Eighth, in our study, the PPI was documented without disrupting the triage workflow; nevertheless, we acknowledge that ensuring waveform quality, permitting a short stabilization period, and maintaining measurement reliability may impose an additional strain on triage personnel in high-volume environments. Lastly, although we found that the PPI had a little better performance than the ESI in predicting discharge, integration of the PPI into real-time triage algorithms was not tested prospectively.

## 5. Conclusions

Although the PPI demonstrates potential as an early indicator of clinical decline, its most practical and significant use may occur during the immediate post-triage assessment or as part of a continuous therapy response evaluation. Our findings indicate that the PPI offers supplementary prognostic information on patient risk. This function may enhance decision-making regarding disposition, escalation of care, or the necessity for more observation. Moreover, its rapid, non-invasive acquisition using existing pulse oximetry platforms ensures that implementation would be cost-effective and scalable. Although this strategy could have potential merit, we did not assess the direct incorporation of the PPI into triage decisions. The impact of the PPI, eventually integrated into composite triage scores, such the ESI, on patient-centered outcomes and ED efficiency might be validated in prospective multicenter cohorts with different patient demographics and healthcare structures.

## Figures and Tables

**Table 1:** Demographic and clinical profiles of the patients.

Parameter	n = 2539
Age, years	47 (31–66)
Gender	
Female	1346 (53.0%)
Male	1193 (47.0%)
Three-Color Scale Triage System	
Red	16 (0.6%)
Yellow	1519 (59.8%)
Green	1004 (39.5%)
ESI Triage System	
ESI 1	22 (0.9%)
ESI 2	616 (24.3%)
ESI 3	638 (25.1%)
ESI 4	620 (24.4%)
ESI 5	643 (25.3%)
Vital Parameters	
Systolic Blood Pressure, mmHg	124 (112–138)
Diastolic Blood Pressure, mmHg	78 (70–85)
Heart Rate, bpm	90 (78–100)
Oxygen Saturation, %	98 (96–99)
Respiratory Rate, /min	21 (20–21)
Body Temperature, °C	36.7 (36.4–37.0)
Peripheral Perfusion Index	3.60 (2.60–4.70)
GCS	15 (15–15)
Emergency Department Disposition	
Discharged	2084 (82.1%)
Admitted to ward	331 (13.0%)
Admitted to intensive care unit	124 (4.9%)
Survival	2521 (99.3%)
Mortality	18 (0.7%)

Legend: ESI: Emergency Severity Index, GCS: Glasgow Coma Scale. Results are presented as median (interquartile range, IQR) and n (%).

**Table 2:** Relationship Between PPI Values, ESI Triage Levels, and Clinical Outcomes.

Parameter	Peripheral Perfusion Index (PPI), Median (IQR:25–75)	*p*-Value
ESI triage system		<0.001 *
ESI 1 (n = 22)	0.80 (0.60–0.87)	
ESI 2 (n = 616)	2.10 (1.80–2.30)	
ESI 3 (n = 638)	3.10 (3.00–3.40)	
ESI 4 (n = 620)	4.10 (3.90–4.40)	
ESI 5 (n = 643)	5.70 (5.30–6.50)	
Emergency Department Disposition		<0.001 *
Discharged	4.00 (3.10–5.10)	
Admitted to ward	2.10 (1.80–2.40)	
Admitted to intensive care unit	1.90 (1.60–2.20)	
Discharged	4.00 (3.10–5.10)	<0.001 **
Admitted	2.00 (1.70–2.40)	
Mortality	1.15 (0.80–1.67)	<0.001 **
Survival	3.60 (2.60–4.70)	

Legend: * Kruskal–Wallis test. ** Mann–Whitney U test.

**Table 3:** Cut-off value of the PPI for predicting emergency department discharge.

	Cut-Off	AUC (95% Cl)	SEN (95% Cl)	SPE (95% Cl)	PPV (95% Cl)	NPV (95% Cl)	+LR (95% Cl)	−LR (95% Cl)
PPI	≥2.7	0.926 (0.914–0.937)	0.87 (0.85–0.88)	0.85 (0.82–0.88)	0.96 (0.95–0.97)	0.58 (0.56–0.61)	5.89 (4.72–7.35)	0.16 (0.14–0.18)
ESI	≥3	0.903 (0.890–0.915)	0.88 (0.86–0.89)	0.84 (0.80–0.87)	0.96 (0.95–0.97)	0.60 (0.57–0.63)	5.55 (4.49–6.86)	0.15 (0.13–0.17)

Legend: PPI: Peripheral Perfusion Index, AUC: area under the curve, SEN: Sensitivity, SPE: Specificity, PPV: Positive predictive value, NPV: Negative predictive value, +LR: Positive likelihood ratio, −LR: Negative likelihood ratio.

**Table 4:** Correlation between vital parameters and PPI.

Parameter	Peripheral Perfusion Index (PPI)	
	rho	*p* Value *
Systolic Blood Pressure (SBP)	−0.071	<0.001
Diastolic Blood Pressure (DBP)	0.050	0.012
Heart Rate (HR)	−0.018	0.368
Oxygen Saturation (SpO_2_)	0.245	<0.001
Respiratory Rate (RR)	0.116	<0.001

Legend: * Spearman correlation test.

## Data Availability

The datasets generated and/or analyzed during the current study are available from the corresponding author on reasonable request.

## Supplementary Materials

The following supporting information can be downloaded at https://www.mdpi.com/article/10.3390/jcm14134616/s1, Table S1: Logistic Regression Results.

## Abbreviations

The following abbreviations are used in this manuscript:

AUC	Area Under the Curve
CI	Confidence Interval
DBP	Diastolic Blood Pressure
ED	Emergency Department
ESI	Emergency Severity Index
GCS	Glasgow Coma Scale
HR	Heart Rate
ICC	Intraclass Correlation Coefficient
IQR	Interquartile Range
LD	Linear Dichroism
LR	Likelihood Ratio
NPV	Negative Predictive Value
PPI	Peripheral Perfusion Index
PPV	Positive Predictive Value
ROC	Receiver Operating Characteristic
RR	Respiratory Rate
SBP	Systolic Blood Pressure
SpO_2_	Oxygen
