# Supplementary material for: Peripheral Perfusion Index: An Adjunct for the ED Triage or a Powerful Objective Tool to Predict Patient Outcomes?
Source: J Clin Med. 2025 Jun 29;14(13):4616. doi: 10.3390/jcm14134616 (PMC12251100; doi:10.3390/jcm14134616)
Supplement: Supplementary file 1 [file jcm-14-04616-s001.zip › jcm-3641471-supplementary.pdf]

Table S1. Logistic Regression Results

Variable	Coefficient	Standard Error	Z-Value	p-Value	95% CI Lower	95% CI Upper	Odds Ratio (OR)	OR 95% CI Lower	OR 95% CI Upper
Intercept	13.01	38.2	0.3406	0.7334	-61.86	87.88	4.478e+05	1.363e-27	1.471e+38
Peripheral Perfusion Index (PPI)	-17.23	3.141	-5.484	4.149e-08	-23.38	-11.07	3.3e-08	6.997e-11	1.557e-05
Age (years)	-0.06016	0.03762	-1.599	0.1098	-0.1339	0.01357	0.9416	0.8747	1.014
Sex (1=Male)	-0.5951	0.7308	-0.8143	0.4155	-2.027	0.8373	0.5515	0.1317	2.31
Heart Rate (bpm)	0.02889	0.03536	0.8168	0.414	-0.0404	0.0982	1.029	0.9604	1.103
Systolic Blood Pressure (mmHg)	0.01467	0.03678	0.3988	0.69	-0.0574	0.08677	1.015	0.9442	1.091
Oxygen Saturation (%)	0.3222	0.3941	0.8176	0.4136	-0.4502	1.095	1.38	0.6375	2.988
